# “Managed competition” for Ireland? The single versus multiple payer debate

**DOI:** 10.1186/1472-6963-14-442

**Published:** 2014-09-26

**Authors:** Misja Mikkers, Padhraig Ryan

**Affiliations:** NZa, Dutch Healthcare Authority, Newtonlaan 1, Utrecht, The Netherlands; Free University of Amsterdam, Amsterdam, Netherlands; Tilburg University, Tilburg, Netherlands; Centre for Health Policy and Management, Trinity College Dublin, 3-4 Foster Place, Dublin 2, Ireland; Insurance Supervision, Central Bank of Ireland, Dublin 1, Ireland

**Keywords:** Managed competition, Quality, Performance measurement, Market structure, Financing reform, Ireland, Netherlands, Health insurance

## Abstract

**Background:**

A persistent feature of international health policy debate is whether a single-payer or multiple-payer system can offer superior performance. In Ireland, a major reform proposal is the introduction of ‘managed competition’ based on the recent reforms in the Netherlands, which would replace many functions of Ireland’s public payer with a system of competing health insurers from 2016. This article debates whether Ireland meets the preconditions for effective managed competition, and whether the government should implement the reform according to its stated timeline. We support our arguments by discussing the functioning of the Dutch and Irish systems.

**Discussion:**

Although Ireland currently lacks key preconditions for effective implementation, the Dutch experience demonstrates that some of these can be implemented over time, such as a more rigorous risk equalization system. A fundamental problem may be Ireland’s sparse hospital distribution. This may increase the market power of hospitals and weaken insurers’ ability to exclude inefficient or poor quality hospitals from contracts, leading to unwarranted spending growth. To mitigate this, the government proposes to introduce a system of price caps for hospital services.

The Dutch system of competition is still in transition and it is premature to judge its success. The new system may have catalyzed increased transparency regarding clinical performance, but outcome measurement remains crude. A multi-payer environment creates some disincentives for quality improvement, one of which is free-riding by insurers on their rivals’ quality investments. If a Dutch insurer invests in improving hospital quality, hospitals will probably offer equivalent quality to consumers enrolled with other insurance companies. This enhances equity, but may weaken incentives for improvement. Consequently the Irish government, rather than insurers, may need to assume responsibility for investing in clinical quality. Plans are in place to assure consumers of free choice of insurer, but a key concern is a potential shortfall of institutional capacity to regulate managed competition.

**Summary:**

Managed competition requires a long transition period and the requisite preconditions are not yet in place. The Irish government should refrain from introducing managed competition until sufficient preconditions are in place to allow effective performance.

## Background: The Irish and Dutch health systems

Ireland’s general election of 2011 signified a major change in the political landscape. After spending only four of the preceding fifteen years in government, the Fine Gael political party assumed power as the dominant partner in a coalition with over two thirds of parliamentary seats [1]. This electoral mandate gave impetus to Fine Gael’s proposed introduction of ‘managed competition’ between insurance companies, a universal health insurance model based on key features of the Dutch system [2, 3].

Government policy documents set out a three phase plan for reform. The first phase aimed to abolish out-of-pocket payments for primary care for all citizens, however efforts to date have been unsuccessful. The second phase is the introduction of activity-based payment for hospitals, in order to enhance efficiency and alleviate waiting lists, and this is currently underway. The final and most far-reaching phase is the introduction of managed competition, scheduled for the 2016 to 2019 period. This paper debates whether the preconditions for effective managed competition are in place in Ireland. If key preconditions are absent there is a case for the government refraining from this reform or from its stated timeline. Competition between payers is proposed also for England [4] and China [5], therefore this debate is relevant for policy makers in many settings.

This section describes key features of the Irish and Dutch health systems and the rationale for the proposed reform. In Ireland, most health financing derives from statutory resources and flows through a public purchaser and provider, the Health Service Executive (HSE). As a public monopoly, the HSE is not exposed to competition. The proportion of the population enrolled in private health insurance decreased from 48% to 43%, between 2007 and 2012 [6]. Spending on private health insurance amounted to around 13% of health spending in 2010 [7, 8]. Around 10 - 15% of health spending is from out-of-pocket payments. This institutional landscape would be reshaped by managed competition, where insurers would assume most responsibility for financing and purchasing.

The Irish government cites multiple reasons for emulating a ‘Dutch’ model. One reason is equity, as around half of the population is enrolled in supplementary insurance schemes, which currently entitles them to faster access to some essential hospital services [9]. Official policy documents describe the Irish system as “unfair” and “two-tier” [2]. Fine Gael argues also that the Dutch model can alleviate “inefficient” service provision. Clinical quality improvement receives less explicit policy attention, but is a key health system goal [10].

**Figure 1:**
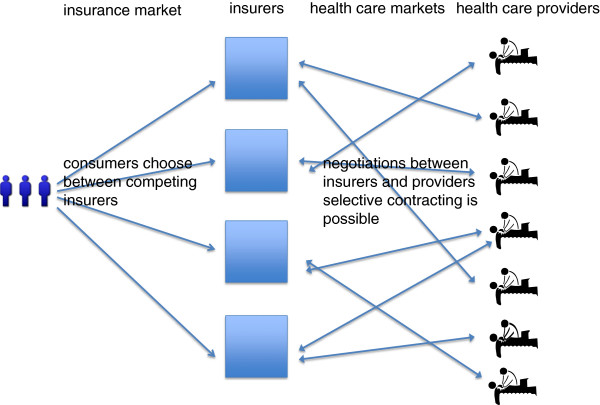
**The idea behind managed competition.**

A tenet of the Dutch model of managed competition is consumer choice of purchaser. Consumers may switch to a rival insurer to seek better price of insurance, service, or value of the underlying provider network. Since market failures plague the health care sector [11], regulation counteracts some of the perverse incentives of traditional health insurance markets. The aim of this model is to align the commercial interests of insurers with consumers’ health and financial wellbeing, whereby insurers can prosper by prudently purchasing health care for consumers [12, 13]. A detailed history of multiple and single payer models is beyond the scope of this paper. The organizational structure of managed competition is depicted in Figure 1.

Key features of the Dutch system include mandatory insurance for all citizens, a standardized benefits package, risk equalization, and competition between insurers to attract consumers. The government defines the benefits package to prevent risk selection and encourage competition on price and quality, rather than on benefit design and market segmentation [14]. Benefits include practically all primary medical care and hospital care, but exclude dental and nursing home care^a^. Supplementary insurance policies including dental and cosmetic care are optionally available. Each insurer’s revenues comprise an annual community-rated premium paid by enrollees, combined with a weighted payment from a risk-equalization fund to reflect individual patients’ risk profile. This dampens incentives to preferentially enroll low-risk patients with predictably lower health care expenses. The system involves virtually no co-payments, a mandatory deductible of € 350 Euro^b^ and an optional deductible (between € 0 and € 500)^c^.

The successful implementation of managed competition depends on a number of preconditions [4]. Moreover, as health systems are a product of country-specific institutional, cultural and socio-demographic factors, an effective model in one setting may be inappropriate elsewhere. Therefore it is important to debate the preparedness of the Irish healthcare system for the introduction of managed competition. This debate mostly concerns the Dutch model and its lessons for Ireland, but we also seek to draw generalizable lessons for other systems. The debate describes successes and failures of the model in the Netherlands, and considers whether the insurer, provider market, and regulatory institution context in Ireland would lead to comparable outcomes.

While this is not a systematic review, we draw on a review of international experience of market competition in healthcare conducted by Gaynor and Town [15]. In addition, in some instances there is limited data on the experience of insurer competition in the Netherlands, and it is appropriate to refer to the experience of the United States, due to its significant experience in regulation of health insurance markets. However, for the most part the debate draws solely on experiences of the Netherlands and Ireland, as findings from other settings are likely to have limited generalizability.

A number of conceptual frameworks are potentially applicable to this debate. Glied [16] specifies three market failures any system must address (industry competitiveness, asymmetric information about health risks and moral hazard and information about health care quality). Okma and Crivelli [17] argue that managed competition should fulfill four assumptions, such as the desire of consumers to actively “shop around” for high value insurance plans [17]. For this debate we adopt the more specific framework of Bevan and van de Ven [4]. Their framework specifies eight preconditions that encompass both [16] and [17].

1. risk equalization
2. market regulation
3. transparency
4. consumer information
5. freedom to contract
6. consumer choice of insurer
7. financial incentives for efficiency
8. sufficient providers and insurers

We shall discuss these items in a slightly different order. The focus of section “Freedom to contract” is the freedom of insurers to contract with providers, and section“Sufficient providers and insurers” deals with the sufficiency of provider and insurer supply. Section“Consumer choice of insurer” addresses consumer choice of insurer, and section “Financial incentives for efficiency” deals with financial incentives for efficiency. Sections “Risk equalization”, ‘Transparency and consumer information’, and “Market regulation” deal with risk equalization, the implications of transparency for clinical quality, and institutions for market regulation respectively. Each section ends by summarizing the fulfillment of these preconditions. A concluding section draws some general inferences from this debate.

## Discussion

### Freedom to contract

The idea behind managed competition is that insurers negotiate with providers on behalf of consumers. Insurers should be free to engage in selective contracting (where they exclude inefficient or poor quality providers from contracts) and to negotiate all contract dimensions (e.g. price, quality and volume). Bevan and van de Ven [4] mention that prices in the Dutch hospital sector were liberalized gradually from 10% of hospital production in 2006 to over 30% in 2009. After their article was published, in 2012, the liberalized part was extended to all elective care (roughly 70% of hospital production). Furthermore, in some other sectors such as physiotherapy and pharmacy, prices are liberalized. In other sectors (e.g. speech therapy) maximum tariffs apply, but competition is fierce and prices are below maximum tariffs [18]. In section ‘The hospital market’ we shall further elaborate on the experience with selective contracting in the hospital market.

In Ireland the freedom to contract will be limited by government regulation, although the details of this are unclear. The claims expenses of insurers will be subject to a cap, which may potentially result in rationing of care and increase the importance of supplementary insurance. The expenditure cap would be based on an insurer’s risk profile and would consider switching between insurers and the rate of healthcare price inflation. The government also proposes to introduce a price cap for individual services, meaning that insurers could negotiate a price below this but could not exceed it.

The propensity for selective contracting in the reformed Irish system is unclear. Under the current system of supplementary insurance, insurers are free to contract selectively [19], and one hospital in an urban area was forced into closure after a major insurer excluded it from its contracts due to alleged high prices. Despite political pressure for State financial support for the hospital, the government did not intervene. Therefore there is precedent for selective contracting in Ireland, but the extent to which this will be permitted under managed competition is unclear [20].

The extent of selective contracting would be influenced by the Irish regulatory framework, and as we discuss in Section ‘Market regulation’, the institutional framework for regulation of managed competition is in an early stage of development. Cultural factors may also influence the likelihood of selective contracting, such as the willingness of patients to travel to access hospital services and the degree of trust in the motives of insurance companies. An analysis of potentially relevant cultural factors is beyond the scope of this debate.

In the Netherlands, insurers’ freedom to contract has increased gradually, and selective contracting is permitted^d^. In some sectors maximum tariffs apply. But since hospital cost is the largest component of healthcare cost, we tend to conclude that this condition is fulfilled in the Netherlands. The propensity for selective contracting in Ireland under managed competition is not yet clear. If the freedom of insurers to contract selectively is hampered, this will obstruct another of our preconditions: incentives for efficiency (Section ‘Financial incentives for efficiency’), illustrating the interlinked nature of some preconditions. Freedom to contract will not be achieved in Ireland because of the presence of price caps. But in light of the peculiar market structure of Irish hospitals, the adoption of price caps for hospital services may be an appropriate lever to achieve efficiency, as we describe in the following section.

### Sufficient providers and insurers

A health system consists of multiple interlinked markets. Even if managed competition achieves exceptional competition in the market for insurance, insurers must be powerful enough to extract value from the provider market. This section first describes the competitiveness of health insurance markets in the Netherlands and Ireland, and explores the implications for efficiency and quality. It then explores the influence of hospital market structure on insurers’ ability to prudently purchase care, and the consequences for fulfillment of this precondition in Ireland.

#### The insurance market

The degree of competition between insurers influences their performance, although not in a uniform manner. Four private health insurers operate in Ireland, offering coverage for supplementary and some complementary services (such as dental and travel insurance) to almost 50% of the population. The largest insurer, VHI, had 60% market share as of July 2011, followed by Quinn Healthcare (now Laya Healthcare) and Aviva Health at 22% and 17.8% respectively, with restricted membership undertakings accounting for the remaining insured population (e.g. related to employment) (HIA 2011). The VHI operated as a state supported monopoly from 1957 until the entry of a rival (BUPA) in 1997, followed by additional entrants in 2004 and 2012.

Competition agencies often calculate the Herfindahl-Hirschman Index (HHI) as a measure of market concentration. The HHI can be interpreted as an average market share, ranging from slightly above zero for atomistic market shares to 10,000 for a monopolist. Between 1,500 and 2,500 can be considered moderately concentrated (see for example the Horizontal Merger Guidelines of the U.S. department of Justice and the Federal Trade Commission [21]). Since insurance can be conceptualized as an option to access provider markets that are typically local or regional, insurance markets should be determined on a local or regional level [22].

Regional data are unavailable for Ireland. The national HHI for Ireland’s insurance market in July 2011 was approximately 0.442, suggesting quite little competition. Indeed, profits for one insurer reportedly amounted to 17.3% of premiums in 2004 [23], and more recent analysis suggests persistent inefficiency and poor contracting practices in one major insurer [24]. The number of insurance products on the market increased from 18 in 2003 to over 200 by 2012, and this may have facilitated widening risk profiles between insurers [23]. There has been significant potential for insurers to compete on the basis of risk selection rather than extracting value from providers (see Section ‘Risk equalization’).

European competition law prohibits public subsidization of an insurer in a competitive market [25]. The Irish government initially aspired to design a system exempt from European and national competition law [3], but recently reversed this stance and stated that competition law shall apply [19]. Ireland’s proposed system would incorporate a public insurer, and if this occurs on a ‘level playing field’ without any special support for the public insurer, this may have little bearing on dynamics and incentives. But if risk-equalization is weak and the government subsidizes the public insurer, it could become burdened with costly patients (such as the chronically ill) while private insurers risk-select more profitable patients.

**Figure 2:**
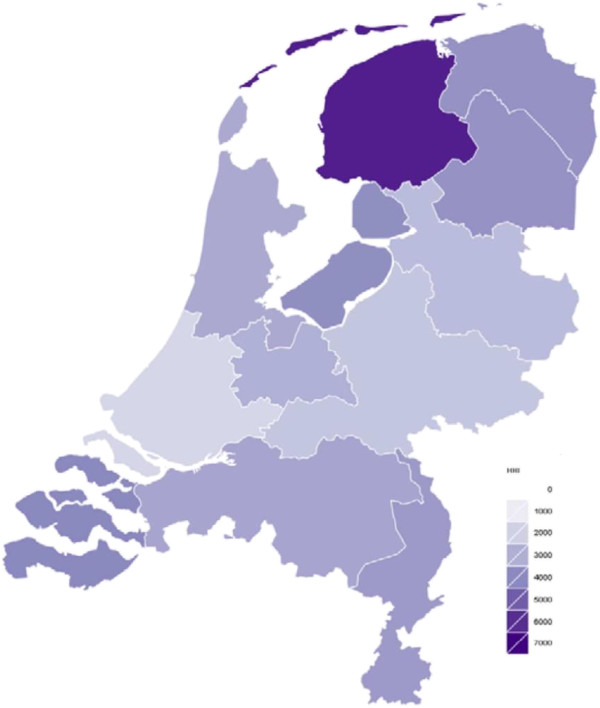
**An overview of the HHI per province in the Netherlands** ^**e**^ **.**

In the Netherlands, the insurance market structure has been dynamic since 2006, partly characterized by mergers that increased HHI. The market is fairly concentrated, and the national market shares of the four largest insurers ranged from 13% to 33% in 2012. As shown in Figure 2, if we consider a market with an HHI larger than 2,000 to be concentrated, all but one (South Holland) of the provincial insurance markets are concentrated [26]. The northern province of Friesland even has an HHI of above 7,000. However, since the products of the insurers are relatively homogenous (with respect to the benefits package and the underlying provider network), even a few firms may engage in fierce competition. An indication of this might be insurers’ annual loss of €600 million on the standard benefit package during the initial years after reform [27]. Since 2009 insurers have made an annual profit on the basic package. In particular, profits increased in 2012 because of cheaper purchasing of pharmaceuticals [28].

Determining the appropriate number of insurers poses trade-offs. On the one hand, more insurers potentially lead to greater price competition and better value for consumers. As shown by a recent review [15], most evidence comes from markets in the United States (US). One of the more sophisticated analyses examined insurers’ ability to price discriminate employers based on their profit level, and although the analysis did not directly assess market power, the findings suggest that markets with fewer than seven insurance companies are not competitive [29]. The benefits package is not homogeneous in the US therefore it is difficult to generalize this finding.

On the other hand, mergers between insurers may enhance performance in multiple ways. First, a larger firm may experience efficiencies of scale due to superior risk pooling and spreading of administration costs. Second, larger insurers may negotiate better prices from health care providers. Dutch insurers have argued that mergers were intended to boost negotiating power vis-a-vis hospitals [30], supported by a negative correlation between insurance market concentration and hospitals’ price-cost margin [31]. This resonates with findings in other countries [32].

In the third instance, more competition between insurers may give rise to underinvestment in quality, shown empirically by Chernew et al. [33]. When a health insurer invests in quality improvement, providers typically offer the same quality to patients enrolled with rival insurers. Consequently, despite its societal benefits, quality improvement may offer no market advantage over rival insurers, which discourages investment by insurers. Section ‘Transparency and consumer information’ examines incentives for clinical quality in more detail.

#### The hospital market

The number of hospitals influences the dynamics of insurer-hospital negotiations, which in turn influences hospital efficiency. For societal benefit, insurers should be powerful enough to extract value from providers. This power derives largely from the threat of selective contracting, whereby insurers terminate a provider’s contracts in favor of rival providers offering superior value [34]. Selective contracting may be less viable in Ireland than in the Netherlands, and this could be a key challenge when implementing managed competition.

Markets must contain sufficient providers to enable insurers to contract selectively [4]. Although terminating contracts with a single hospital may have little direct impact on insurance premiums, which depend on the prices of many hospitals, it may enhance insurers’ negotiating power by increasing the credibility of future threats. Conversely, excluding a hospital could theoretically enhance the bargaining power of nearby hospitals by dampening competition.

Despite its importance, selective contracting is uncommon in the Netherlands [35]. Insurers generally contract more than 95% of providers. Many insurers backtracked from initial attempts at selective contracting, due to concerns of reputational damage which could prompt consumers to switch insurers [14]. Dutch citizens have favoured retaining local hospital services [36] and may mistrust insurers who restrict access. Travel time may explain as much as 74% of hospital choice in the Netherlands according to one study [37], although other studies suggest a more modest role (see Section ‘Transparency and consumer information’). Therefore consumers may not (yet) be willing to trade-off local access for a smaller premium.

Dutch legislation forces insurers to contract with a provider network offering certain services (mainly acute care) within a specified distance of each enrollee’s residence. This may further impede selective contracting, creating an accessibility-efficiency trade-off. Moreover, hospitals considered to be pivotal in regional service provision may receive some government protection from bankruptcy, strengthening their market power [38]. As an alternative to selective contracting, some Dutch insurers have experimented with steering patients to preferred providers using positive and negative financial incentives. However the impact of this is unclear [14].

Even if insurers have the freedom to contract, the use of selective contracting may be less viable in Ireland than in the Netherlands. This is due to relative population density. Population density is six times greater in the Netherlands than Ireland^f^, and consequently Dutch hospitals tend to be closer together than Irish hospitals [39], despite the large size of Dutch hospitals by international standards. The landmass of the Republic of Ireland is around 1.67 times that of the Netherlands^f^, and contains 71 acute hospitals (52 public and 19 private), compared to around 93 general hospital organizations and 198 independent treatment centres (the latter providing non-acute surgical interventions) in the Netherlands [40].

There are multiple academic teaching hospitals in Dublin, Ireland’s capital city, but markets in the rest of the country are more concentrated. This could give many hospitals *de facto* local monopoly status. Where hospitals are further apart, *ceteris paribus*, consumers could be less willing to travel beyond a local hospital. An insurer who contracts selectively may lose significant market share, as enrollees may switch to a rival insurer offering local services, and this weakens the credibility of the threat of selective contracting. Robust quality information may attenuate this problem, but this is not available.

Cultural norms may influence the ability of insurers to terminate hospital contracts. Attempted closures of hospitals in Ireland tend to be politically contentious and have elicited public protest [41], suggesting that selective contracting may encounter resistance. A comparison of potentially relevant cultural norms in Ireland and the Netherlands is beyond the scope of this debate. If legislation forces Irish insurers to contract with a network of providers that offers services within a specified distance of enrollees’ residences, this would further hamper selective contracting, constituting an accessibility-efficiency trade-off. Another potential barrier is a shortfall in hospital capacity [9, 42]. But waiting lists may have dissipated to an extent, and one report suggests that in November 2012 less than 500 people were waiting more than nine months for inpatient treatments, compared to over 1,500 people in December 2011 [43].

**Figure 3:**
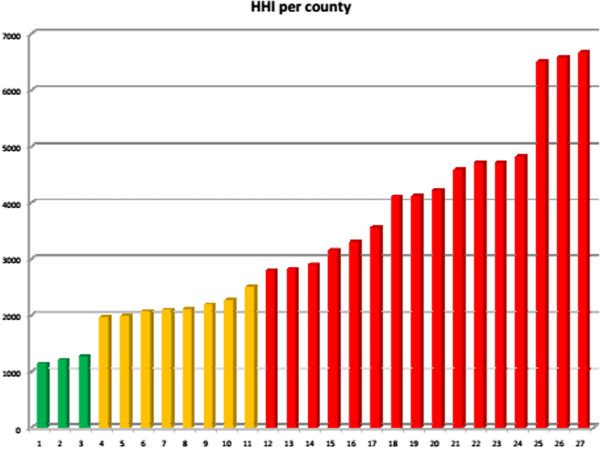
**The HHI per county in Ireland** ^**h**^ **.**

**Figure 4:**
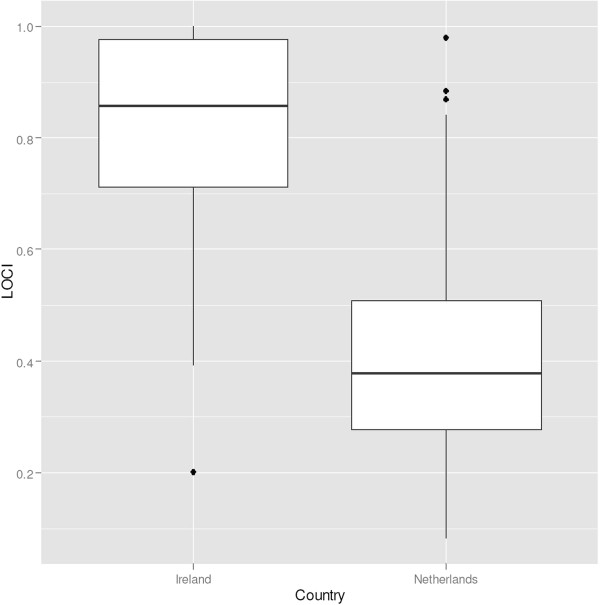
**The Loci in the Netherlands and Ireland based on data 2009** ^**i**^ **.**

Empirically, there is evidence of a positive association between the market shares of hospitals and the hospital price cost margin in the Netherlands [31] and the US [32], while evidence from other settings is limited. An analysis of six hospital mergers in the Netherlands found price increases for hip surgery of up to 16% [44]. In Ireland, if counties are the relevant market, many hospital markets are highly concentrated according to the HHI^g^ (Figure 3). By contrast, analysis of another parameter, the Logit Competition Index (LOCI) [45], suggests that hospital markets in Ireland are les concentrated than the Netherlands. Figure 4 shows the LOCIs for the Netherlands and for Irish public hospitals, where a higher LOCI signifies greater competition, ceteris paribus. The Dutch data in Figure 4 is based on unpublished observations from the Dutch Healthcare Authority. Results are difficult to interpret due to lack of data on Irish private hospitals, and as capacity shortfalls may force patients to travel for services. Competition may be limited even for some high volume DRGs, as activity tends to be concentrated in a few Irish hospitals.

To conclude, while in the Netherlands the insurance market looks concentrated, the conclusion is that the insurance market is relatively competitive [46]. The reason is that in markets with homogeneous products (as in the Netherlands) less market players are needed to compete. The insurance market in Ireland is also concentrated. However, if a universal benefits package will be adopted in Ireland, competition between insurers may increase. Moreover, there are other potential benefits to having a market with larger insurers, as they may be less likely to under invest in quality and may negotiate better prices. Our conclusion, based on the current working of the Dutch system, is that the market structure of the insurance market is not an obstacle for the introduction of managed competition in Ireland.

**Figure 5:**
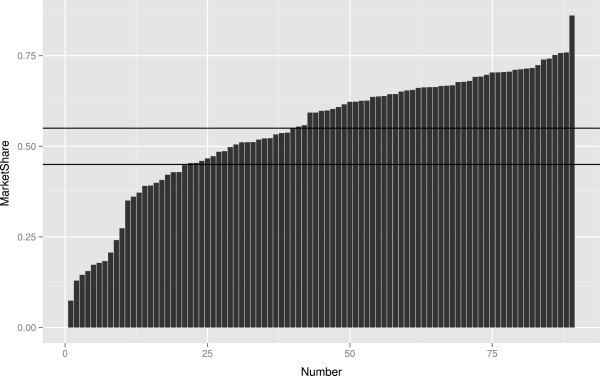
**Market shares of hospitals in the Netherlands.**

The other requirement is a sufficient amount of providers. The Dutch experience teaches us that in many sectors (such as physiotherapy, speech therapy, pharmacies), there are sufficient providers. These providers have claimed that insurers possess excessive monopsony power [18]. We have focused on the hospital market, as this sector is responsible for most of the expenses of insurers. The hospital market in the Netherlands is concentrated as many firms have a market share of above 55%^j^. In Ireland, the relatively large distances between hospitals may inhibit insurers’ ability to contract selectively, and this may be one of the less tractable problems facing managed competition. Figure 5 presents the market shares of hospitals in the Netherlands, based on unpublished observations from the Dutch Healthcare Authority.

## Consumer choice of insurer

In managed competition, insurers compete for contracts with consumers. To fulfill this condition consumers should have a free choice of insurer without encountering high transaction costs. As Bevan and van de Ven [4] conclude, consumers in the Netherlands have choice and insurers are obliged to accept any consumer without price discrimination.

In the Netherlands, annual switching rates between insurers fell from a peak of 20% in 2006 to around 3–4% per year during 2008 and 2009, then increased to 8,3% in 2013 [28]. Some analysts interpret low rates of switching as a lack of competition [17]. However, low switching rates may also signify a competitive market in which quality and efficiency are improving. It is concerning that the elderly and chronically ill may be deterred from switching, due to fears their new insurer will not enroll them in voluntary supplementary health insurance [4].

The Irish government stated that consumers will be permitted to choose an insurer annually, and that insurers will not be permitted to reject them (open enrollment). In addition, consumers will be permitted to renew their cover for the duration of their lifetime, and insurers cannot price discriminate on the basis of age or morbidity (community rating) [19]. However, the enforcement of these objectives is dependent on the creation of sufficient regulatory capacity, which we discuss in section ‘Market regulation’. Cultural norms may also influence the degree to which insurance companies adhere to these principles, and the degree to which deviant behaviour by insurers is tolerated, however a detailed discussion of cultural norms is beyond the scope of this debate.

In sum, as Bevan and van de Ven [4] conclude, this condition is fulfilled in the Netherlands. The Irish government aims explicitly to fulfill this condition from the outset of managed competition. Section ‘Market regulation’ describes the Irish plans to ensure an appropriate regulatory framework is in place to achieve this.

## Financial incentives for efficiency

Managed competition seeks to achieve value by reducing waste in the delivery of care. In the Netherlands, price growth in the liberalized (insurer negotiated) segment of hospital services has been slower than in the price-regulated segment since 2006 [14], potentially due to insurer competition and negotiating power. Increased activity levels that resulted in shorter waiting lists, have also counterbalanced any savings in unit costs.

If activity increases are meeting previously unmet need in a cost-effective manner, this is a favorable outcome. But the additional activity may reflect some supplier-induced demand, a form of unnecessary treatment [47]. Unnecessary treatment may be widespread, due to the frequently subjective nature of clinical need, and failure to match care patterns with patients’ individualized preferences [48].

Some insurers seek to temper volume growth by specifying volume caps in hospital negotiations, such as in the form of lower prices beyond a certain volume threshold. A weakness of the system, described by Bevan and van de Ven [4], has been the complex, unwieldy classification scheme on which hospital payment is based. This comprised over 30,000 unique diagnosis treatment combinations, and although the scheme has been simplified it remains a work in progress.

**Figure 6:**
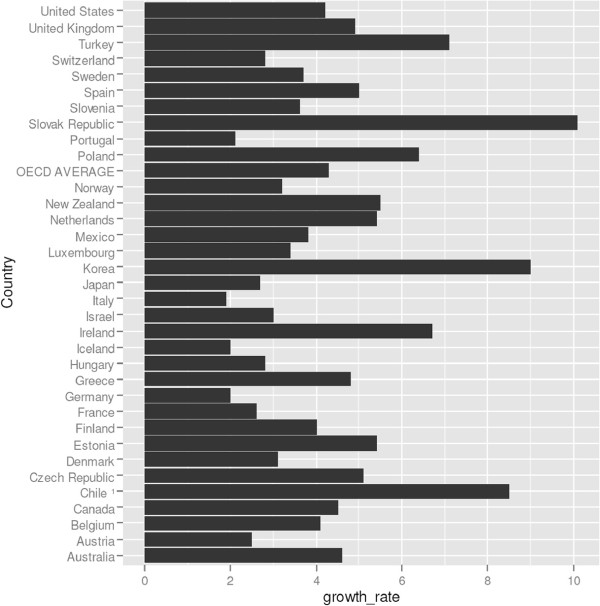
**Cost growth in OECD countries, average annual growth of total expenditure on health in the period 2000–2010 or nearest year, in real terms** ^**k**^ **.**

It is unclear whether managed competition is more or less susceptible to wasteful spending than a single payer framework. Looking at cost growth in OECD countries (Figure 6), it is not immediately apparent whether countries with multiple payers perform better or worse than single payer countries. For example the annual growth rate of total expenditure on health in real terms of the US is slightly lower than the OECD average, while the annual growth in single payer countries like Australia, Spain, Ireland and the UK is higher. On the other hand the annual growth in single payer countries like France, Norway and Sweden are lower than in the US.

**Figure 7:**
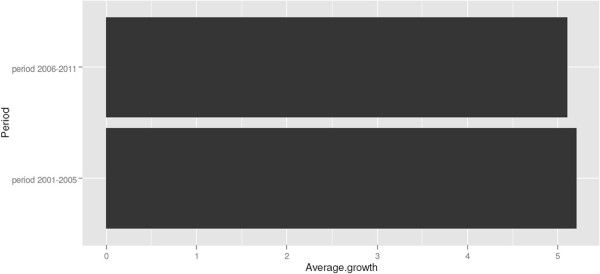
**Average annual cost growth of hospitals and medical specialists in the Netherlands, based on data from the Dutch Statistical Bureau (CBS)** ^**l**^ **.**

Figure 7 shows the growth rate in hospital spending in the Netherlands in the period before rate deregulation (2001–2005) and after deregulation began (2006–2011). The average annual growth rates between the two periods are virtually indistinguishable

In Ireland, empirical analysis suggested that around 19% of spending in hospitals is wasted due to operational inefficiency. This analysis did not consider quality, and it omitted potential savings from lower input prices and from transfer of care to lower-cost settings such as primary care [49]. Inefficiency appears to be a contributing factor to the lengthy waiting lists encountered for many hospital services.

If managed competition is to mitigate this inefficiency in Ireland, a robust system of contracting should instill appropriate incentives. Currently, hospitals are predominantly paid by incremental budgeting related to historic spending patterns, with modest activity based payment adjustments [49]. Hospital efficiency is assessed for activity accounting for 80% of hospital budgets, yet payment adjustments are a maximum of 3% of budgets [50, 51]. A transition towards increased activity-based payment is currently underway [52].

The efficiency of health care providers is a function of financial and intrinsic incentives. In order to respond optimally to financial incentives, providers must possess managerial expertise and a culture that facilitates improvement. There has been limited systematic effort to improve quality and efficiency in Irish care delivery, and there is potentially scope to improve the cultural attitudes of clinicians towards care redesign for efficiency and high quality [10]. However, it is unclear how these cultural factors in Ireland compare to the Netherlands.

The issue of efficiency is intimately linked to some other preconditions. The power to contract selectively (freedom to contract) and the number of providers both influence insurers’ negotiating power, with direct implications for efficiency. If the government proceeds to introduce price caps on hospital services, this could also facilitate efficiency. The rigour of risk equalization and regulation are other key factors. Therefore these preconditions can be viewed as mutually dependent, rather than as isolated issues.

In sum, there is room to improve incentives for efficiency in both countries. Yet deficient incentives would also hamper the performance of a single payer system. Therefore, this is not necessarily grounds to postpone the introduction of managed competition, provided that its cost control mechanisms are at least as effective as a single payer system.

## Risk equalization

Managed competition attempts to align the interests of insurers and patients. Ideally, insurance companies would prosper by extracting value from providers, and consumers’ choice of insurer would lead to an efficient allocation of resources. However health insurance markets are prone to information asymmetries, often leading to market failure and efficiency losses [11]. Managed competition seeks to mitigate this problem.

Adverse selection refers to patients using private information to gain advantage in insurance markets. It may also refer to the use of private information by insurers to gain advantage, which we address subsequently in the paragraphs on risk selection. Consumers expecting high health care costs often choose more expensive and generous plans, while those expecting low costs may prefer cheaper plans. When this differential selection occurs because price does not reflect an individual’s marginal cost, this is adverse selection. An efficiency loss arises because of the difference between price and cost [53].

Adverse selection results in some loss of desirable risk spreading within plans, due to relatively healthy enrollees opting out and sicker patients remaining enrolled. The mean cost per enrollee increases in generous plans, and sick people end up paying larger premiums than healthy people. In its extreme form this results in unsustainable price and cost increases, and the potential termination of a plan, known as an “adverse selection death spiral” [53].

The Dutch and Irish insurance systems share some common strategies to mitigate information asymmetries. Each system is based on open enrollment, meaning insurers cannot refuse to insure anyone based on their health risk. This weakens insurers’ ability to enroll profitable consumers with a favorable risk profile or to prevent enrollment of predictably lossmaking consumers risk-select, collectively referred to as risk selection. However in Ireland there are waiting periods for coverage in some cases, such as after upgrading to a more generous plan. Each insurance system is based on community rating, whereby enrollees in a plan pay identical premiums irrespective of individual risk.

Community rating prevents price discrimination, but simultaneously strengthens insurers’ incentives for risk-selection. By contrast, under experience rating, insurers would set individual premiums commensurate to individual risk [23].

The Dutch system mitigates concerns of adverse selection by standardizing the basic benefits package and mandating insurance for each citizen. Therefore insurers cannot strategically modify their basic offerings to attract favorable risks. The Irish insurance market has been quite vulnerable to adverse selection, firstly because insurers must cover a minimum level of benefits rather than standardized benefits. Some policies offer reduced premiums but restrict the benefits level for orthopaedic and ophthalmic procedures, which may attract younger enrollees [54].

Secondly, premiums in Ireland are community rated with limited cross-subsidization to reflect risk profile differences. This cross-subsidization is known as risk adjustment or risk equalization, and according to Bevan and van de Ven [4] this is the first precondition for successful managed competition. As Arrow [11] noted, in a ‘genuinely’ competitive market the only possible income redistribution by premiums is that which reflects differences in risk profiles. Differential selection into Irish plans may have contributed to annual price increases of up to 45% for policies held mostly by older people. For a more detailed discussion of community rating in Ireland, see Turner and Shinnick [23].

The Dutch model uses sophisticated risk equalization to dampen incentives for risk selection. Therefore insurers offering high quality chronic disease management are not penalized for attracting more expensive consumers with multiple chronic diseases [55, 56]. By contrast, risk adjustment in the US has been limited, and one study found that eight of ten plans understated the generosity of their mental health benefits, reportedly to avoid attracting loss-making risks [53].

In the Netherlands, an ex-ante (prospective) system compensates insurers for actuarially predictable health expenditure differentials induced by socio-demographic and morbidity variables, such as age, sex, income, location and prior health care consumption (chronic pharmaceutical dependencies and prior hospitalization). This levels the playing field and enables competition on price and quality, rather than on risk profile. Until 2012 an ex-post (retrospective) risk-sharing scheme was in place, and this accounted for an estimated 25% of total risk-equalization payments in 2010 [14]. This reduced incentives for risk selection, but also diluted ex-ante incentives for vigorous price negotiations with providers.

Risk-equalization is technically demanding. It need not be ‘perfect’, but should be sophisticated enough to dissuade insurers from risk-selection. In the Netherlands insurers can identify unprofitable patient subgroups, but there is not yet evidence of negative risk-selection [56]. Among consumers who did not switch insurer during 2011, around 3% cited concern of being rejected for complementary insurance as a reason [57]. There are concerns that complementary health insurance and selective contracting may serve as risk-selection tools [14].

Of note, the ex-ante system incentivizes insurers to monitor the accuracy of risk equalization. Insurers employ skilled econometricians to analyze expenditure patterns and identify risk patterns that benefit rival insurers. If a particular sub-population of enrollees is demonstrably under- (or over-) compensated, the system can be refined to reflect this. This is a budget-neutral system therefore payments not distributed to one insurer are distributed to their rivals, strengthening incentives for vigilant monitoring.

It is the ex-ante risk equalization that gives insurers the incentive to seek efficiency in manage insurance and the delivery of care through contracting [4]. Until 2012, as part of the transition, some ex post compensation for insurers was in place. The ex post mechanisms have been largely abandoned in 2012.

In Ireland, risk-equalization has improved significantly in recent years. Since January 2013 a new risk-equalization algorithm accounts for age (over 60 years, in 5 year bands), gender, the level of plan benefits, and overnight hospital stays in a private or semi-private bed [3, 58]. This follows a setback in 2008 when efforts to introduce risk-equalization accounting for enrollee age, gender and health status were deemed unconstitutional by a Supreme Court ruling [54]. The Court interpreted legislation as permitting community rating within plans but not across multiple plans.

From 2009, a levy system partially compensated insurers with a higher proportion of elderly patients [54]. Another measure was tax relief on a sliding scale for older enrollees (over 60 years in 2011) to partly offset the cost of higher premiums [23]. Tax relief compensated for 65% of the higher costs of older enrollees in 2011, increased from 50% in 2009 and 2010. Therefore incentives for risk-selection remained significant [54]. Further improvements to the risk adjustment algorithm will require rich socio-demographic and clinical data. A national unique identifier number for patients could facilitate data collection, and in 2013 the government published legislation to create a legal basis for its introduction [59]. Authorities are currently developing information governance, technical and management standards to enable implementation of the unique identifier [60].

In sum, the Dutch model demonstrates that robust risk equalization may largely deter risk-selection. Matching the strengths of this model would be vital for managed competition in Ireland. This issue is linked to some other preconditions: if risk-equalization is underdeveloped, this may dampen the incentives for selective contracting and efficiency. Despite significant strengthening of the Irish risk equalization algorithm it remains inferior to the Dutch model, and this should be further enhanced prior to the introduction of managed competition.

## Transparency and consumer information

Transparency is an important attribute of the benefits package, the hospital payment mechanism, and quality of care. We discussed the payment mechanism for hospitals in section ‘Financial incentives for efficiency’. As regards the benefits package, the standardized package in the Netherlands is outlined in the Health Insurance Act, although this does not apply to supplementary health insurance packages. Each insurer must offer the basic package to consumers. In Ireland the benefits package has not been rigorously defined [61], but the government recognizes the importance of transparency and uniformity. The Minister for Health will determine the contents of the benefits package, based on costed scenarios developed by an advisory Commission which has yet to be established. A portion of the benefits package will be provided by the public health service outside of the universal health insurance system [19]. The remainder of this section focuses on transparency of clinical quality and on the incentives of managed competition for quality improvement.

### Choosing quality

High quality care is vital in any single or multiple payer system. In the Netherlands, a retrospective chart review suggested that around 1,700 hospitalized patients may die from avoidable errors each year. An adverse event occurred in 5.7% of all admissions to Dutch hospitals, 40% of which were considered preventable, and 12.8% of which led to permanent disability or death. More than half of adverse events related to surgery [62]. A more recent analysis found that elderly patients are more likely than younger patients to suffer an adverse event in hospital, the consequences are likely to be more severe, and the adverse events are more likely to arise from medication error [63]. There appears to be unwarranted variation in practice patterns across Dutch regions [64].

The research base in Ireland is scant, but we have little grounds to believe outcomes are superior. Ireland’s efforts to measure and improve quality are in their infancy [65]. There are national systems to record adverse clinical incidents and “hospital acquired diagnoses” in public hospitals [66]. However there is no national registry for tracking chronically ill patients, and relative to the United Kingdom or the US there are few initiatives to reduce the rate of clinical errors [10]. As noted in section‘Financial incentives for efficiency’, there may be scope to improve cultural attitudes in Irish provider organizations towards care redesign and quality improvement. However, it is unclear whether there are significant cultural differences between clinical providers in Ireland and the Netherlands.

It is not clear whether a single or multiple payer system achieves better quality. Neither system can achieve high quality in Ireland in the short term. An ideal analysis would compare the likely trajectory of quality under each system, however as an experimental comparison is not feasible, we must base this on fragmented evidence.

By focusing on consumer choice, the pluralistic Dutch institutional environment may have facilitated (or even catalysed) increased performance measurement [14], however it is difficult to isolate the impact of managed competition from secular trends.

The Netherlands has the second highest number of websites (eleven) of any country offering information on health care quality [67], hospitals have been compelled to report standardized quality indicators since 2009 [68], and consumer surveys routinely assess satisfaction with insurers and providers [14]. Average performance on the majority of hospital process and outcome indicators improved over 2004 - 2008, although some clinical metrics deteriorated [68].

But incentives for Dutch insurers to aggressively improve quality are quite weak. Among consumers who switched insurers, around 52% were motivated by price differentials, 4% cited the standard of insurer services, and only 1% cited dissatisfaction with clinical quality in 2011 [57]. Moreover, a majority of patients base hospital choice on physicians’ opinion [69, 70], whereas only around 1.3% used publicly available quality information [70]. One study suggested that travel distance may explain 74% of hospital choices [37], while others found that hospitals’ reputation and travel distance are modestly important [69].

The scant influence of quality is perhaps unsurprisingly given the quite crude nature of existing information [40]. If quality reporting improves, insurers may become more confident in restricting access to low quality providers without disenfranchising consumers [30]. Yet even when robust outcomes information is available, consumers may be unable to rationally compare the quality of care of each insurer. As most consumers are healthy when selecting an insurer, they should weigh up the probability of developing each disease with its associated quality of care. This would probably be impractical and unappealing. Conversely, consumers who are ill may base their choice of insurer on quality indicators for a single condition.

Of note, around 70% of Dutch consumers were insured in group purchasing organizations in 2012. These collective contracts often select an insurer on behalf of consumers. The implications for quality and efficiency are unclear [57, 71].

The increased emphasis on quality in the Netherlands is to be welcomed. However performance measurement is methodologically challenging and public performance reporting may give rise to unintended consequences such as patient selection by providers [72], data misrepresentation to improve perceived performance, and prioritization of narrow, organization-specific objectives to the detriment of wider health system goals [73]. Performance measurement in the Netherlands does not adjust for patients’ underlying risk profile, and hospital rankings exhibit marked variability [14]. Patients with co-morbidities pose particular difficulties for performance measurement, and around 37% of Dutch citizens over 55 years of age have two or more chronic illnesses [74]. Therefore caution is required.

### Free-riding and prevention

A multi-payer environment creates some peculiar disincentives for quality improvement, one of which is free-riding by insurers on each other’s quality investments [75, 76]. If a Dutch insurer invests in improving hospital quality, hospitals will probably offer the same quality standards to consumers enrolled with other insurance companies. This enhances equity [36], but may weaken insurers’ incentives for quality improvement.

Consider the prevention of central line associated bloodstream infections. Prevention is possible by implementing a simple 5 item checklist, and while data are limited for the Netherlands, we know that prevention could avert up to 20,000 fatalities in US hospitals each year while lowering costs by $2 Billion. Free-riding appears to have deterred insurers from investing in preventive strategies [77]. At its worst, free-riding can cause a ‘race to the bottom’ with negligible efforts by insurers to improve quality [75]. In theory, authorities might circumvent free-riding by publishing robust performance information for disease- and intervention-specific patient cohorts. This could inform patient choice and spur improvement, because even if insurers are not differentiated by quality, hospitals could have strong incentives for quality to attract patients.

For historical reasons the Dutch insurance system may not be very susceptible to free-riding. Each regional market tends to be dominated by a single insurer, with one insurer typically contracting around 60–70% of a hospital’s activity [31]. This is a legacy of many insurers’ former status as sickness funds operating as local monopolies. In Ireland, the structure of local insurance markets would influence the extent of thisproblem.

The original proposals for managed competition were based on vertical integration between insurers and providers [78]. This mitigates the free-riding problem, as investments in quality are unlikely to benefit rival insurance companies. There is scant evidence of vertical integration’s effects on market power, but it may harm competition by restricting rivals’ access to key inputs [32]. Sparsely populated areas probably have too few providers for vertical integration by all insurers [79]. In the Netherlands, vertical integration has been rare, and one insurer (Menzis) that owns or pays for some primary care facilities permits their use by non-enrollees [30]. The Dutch Parliament has considered prohibiting integration between hospitals and insurers.

Another perverse incentive relates to preventive care. Evidence on the cost-effectiveness of preventive care is mixed, but some interventions such as smoking cessation may improve wellbeing and curtail expenditure. Managed competition may instill quite weak incentives for prevention of illness, because of the lag between preventive care and the actual prevention of illness. Enrollees may switch to a rival insurer during this lag, allowing a rival insurer to reap the return on investment. The extent of this problem depends on the turnover rate and the characteristics of enrolees who switch. In general, younger and healthier enrollees are most likely to switch [80, 81].

Risk adjustment also exerts perverse effects on preventive care. In the absence of risk adjustment, insurers may incur predictable losses from chronically ill patients, which can create a business case for investing in prevention. But risk adjustment, by compensating insurers for the incremental cost of chronically ill patients, dampens incentives for prevention [82].

To conclude, achieving high quality is problematic in both single and multiple payer systems. A multiple payer environment may facilitate performance measurement and reporting, but poses distinctive problems such as free-riding. The superior lever for quality improvement in Ireland is unclear. Although the disincentives of managed competition for quality improvement are concerning, they are not necessarily grounds to postpone the introduction of managed competition.

Therefore, at present the precondition of a transparent benefits package is not fulfilled in Ireland, and moreover the institutional arrangements to accomplish this are not yet in place.

## Market regulation

The health system is susceptible to numerous market failures. Therefore, according to Bevan and van de Ven [4] any country needs to have four authorities situated at arm’s length from the government to address specific market failures. They mention the Dutch context where a quality authority seeks to protect consumers against “substandard quality of care”, a financial regulator is present to safeguard the solvency of insurers, alongside a competition authority and a sector specific competition authority/regulator^m^.

In Ireland, by contrast, the institutional framework for managed competition is less developed. Some necessary institutions are in operation but their capacity to regulate a system of universal health insurance is unclear. Other key institutions have yet to be established.

According to government documents, a National Insurance Fund will be established to assess consumers’ entitlement to financial support when purchasing a premium, and to administer support payments directly to each insurer on behalf of consumers. Novel processes shall also be created for the resolution of contractual disputes between insurers and providers. Ireland has an established competition authority, but not a sector-specific healthcare competition authority. The body responsible for regulating the health insurance market is the Health Insurance Authority, which has been in operation since 2001 [19].

A key body is the Health Information and Quality Authority (HIQA), established in 2007 with the mandate of enhancing the quality of health and social services. Its statutory responsibilities include creating and enforcing standards for quality, conducting health technology assessments, and publishing performance information. In managed competition, HIQA would be responsible for adjudicating on disputes between purchasers and providers or members of the public, where there is disagreement regarding the interpretation of the contents of the benefits package. It will also assess the value of clinical interventions so as to inform decisions relating to the composition of the package. It is proposed to create a Patient Safety Agency to advocate for patients who wish to make complaints about care quality, which will complement the work of HIQA [19].

In sum, despite the presence of significant regulatory capacity, this precondition is not fulfilled in Ireland. Existing institutions can contribute to key regulatory tasks, but would appear to require significant bolstering of their institutional capacity, and the creation and development of new institutions will take time.

## Summary

The Netherlands, whose journey towards managed competition began over two decades ago, appears to possess most preconditions for effective implementation. There are concerns such as the degree of concentration in the provider market and a lack of transparency about quality, and on balance it is too early to judge the success of the 2006 reform. But the conditions for managed competition appear more auspicious in the Netherlands than in Ireland.

In its favor, although the existing Irish supplementary health insurance market does not appear highly competitive, there may be enough insurers to compete on a homogeneous product (the standardized benefits package). Vigilant antitrust may be needed to prevent excessive concentration of the insurance market. The limitations of the Irish risk equalization system could be resolved over time, provided that sufficient data and technical expertise are available.

It is crucial to develop robust performance measurement and to nurture a culture of clinical improvement, irrespective of the number of payers. The Dutch primary care sector is largely shielded from competitive forces, but appears to offer high quality at low cost [83], illustrating the importance of clinician leadership. A review of international evidence found that competition between hospitals appears to improve quality in markets with administered prices, but the evidence is unclear in pluralistic insurance markets [32]. It is not clear whether a single or multiple payer system would achieve superior quality. Although the Dutch reform is associated with perverse incentives for quality improvement (section ‘Transparency and consumer information’), this is not necessarily an impediment to reform.

A fundamental problem may be a lack of competition between Irish hospitals, due to their relatively sparse geographic distribution, which could weaken insurers’ ability to prudently purchase care. Insurers in the Netherlands have succeeded in reducing the prices of generic pharmaceuticals [47], but have not lowered significantly the average annual cost increase. This is a key dichotomy. When negotiating against stakeholders such as the generic pharmaceutical industry, insurers can extract value through “take it or leave it” offers. Consumers typically are not loyal to generic pharmaceutical companies, and are unlikely to leave their insurance company if they cannot receive medicines from a specific manufacturer. By contrast, consumers’ preference for local hospital services can weaken insurers. Therefore, this divergent cost trajectory for generic pharmaceuticals and hospitals is a predictable effect of market structure. The Irish government appears cognizant of this risk, and proposes the introduction of price caps to mitigate excessive hospital market power.

To summarize, the essential preconditions for managed competition are not yet in place in Ireland, and the reform would require a lengthy transition period with uncertain benefits. But a single payer model, the alternative, is also incapable of optimizing quality and efficiency in the short term.

Key next steps are to develop more robust contracting mechanisms and risk equalization, a legal and technical framework for price caps, and increased autonomy of public hospitals to allow them to retain budget surpluses and thereby strengthen incentives for efficiency [49]. Additional regulatory capacity will be needed, as well as efficiency gains to alleviate under-supply of services. The Irish government should exhibit patience, and refrain from introducing managed competition until sufficient preconditions are in place for effective performance.

## Endnotes

^a^ Long-term care (e.g. nursing homes), and all those treatments and services that cannot be insured individually because such expenses would be too high to bear, notably mental illness requiring prolonged nursing and care, and congenital physical or mental handicap are separately insured under a mandatory one-tier system.

^b^ Increased from 220 Euro to 350 Euro in 2013.

^c^ Deductible: A sum, fixed or variable, specified in an insurance policy, that is deducted from any claim made under that policy; that sum is then paid by the beneficiary, the remainder being paid by the insurer, subject to any to any co-payment, user charge or co-insurance arrangement. (?. Copay- ment: A form of medical cost sharing in a health insurance plan that requires an insured person to pay a fixed dollar amount when a medical service is received. The insurer is responsible for the rest of the reimbursement. Some plans require that a deductible first be met for some specific services before a copayment applies (Interdepartmental Committee on Employment-based Health Insurance Surveys 2002). Interdepartmental Committee on Employment-based Health Insurance Surveys 2002). Definitions of Health Insurance Terms. Washington D.C., Bureau of Labour Statistics. Roberts, J. L. 1998). A glossary of technical terms on the economics and finance of health services. Copenhagen, World Health Organization Regional Office for Europe.

^d^ However there are many court cases and a political debate about the reimbursement if patients go to providers outside the contracted network.

^e^Figure based on [26].^f^United Nations Population Division http://esa.un.org/unpp.^g^In Figure 3 unconcentrated markets are shown in green, moderately concentrated markets are shown in orange and highly concentrated markets are represented in red.^h^Source: The Economic and Social Research Institute. Hospital Inpatient Enquiry Scheme database. http://www.esri.ie (2013).^i^Source: The Economic and Social Research Institute. Hospital Inpatient Enquiry Scheme database. http://www.esri.ie (2013).^j^The Dutch law states it is plausible that hospitals with a market share with a market share above 40% have market power. Hospitals with a market share above 55% are assumed to have market power.^k^Organisation for Economic Cooperation and Development. (2013). “OECD Health Data 2013.” Retrieved Accessed on 5th April 2014, from http://www.oecd.org/health/health-systems/oecdhealthdata.htm (2013).^l^Based on data from CBS (Statistics Netherlands), http://statline.cbs.nl/statweb/dome/?LA=nl.^m^For a more detailed description of the application of competition rules in the healthcare sector, see the following book chapter by Sauter [84].

## Authors’ information

This paper reflects the personal views of the authors, which are not necessarily those of their employers. This paper is not in any way binding for the Dutch government, in particular for future decisions of the Dutch Healthcare Authority on the topics discussed. This paper does not necessarily reflect the views of the Central Bank of Ireland.

## References

[1] Gallagher M, Marsh M (2011). How Ireland Voted 2011: The Full Story of Ireland’s Earthquake Election.

[2] Reilly J: **Faircare: A New Direction for Health Care and Policy in Ireland****.** *Journal of experimental & clinical assisted reproduction* **6** 2009 http://www.jexpclinassistreprod.com/article/view/4601/3195 PMC2868302 20485576

[3] **The Path to Universal Healthcare - Preliminary Paper on Universal Health Insurance** Technical report, Department of Health, Dublin, 2013. http://health.gov.ie/blog/publications/the-path-to-universal-healthcare-preliminary-paper-on-universal-health-insurance/

[4] Bevan G, van de Ven W (2010). **Choice of providers and mutual healthcare purchasers: can the English National Health Service learn from the Dutch reforms?**. Health Econ Pol Law.

[5] Xu W, van de Ven WP (2013). **Consumer choice among mutual healthcare purchasers: a feasible option for china?**. Soc Sci Med.

[6] **Health in Ireland - Key Trends 2013** Technical report, Department of Health, Dublin 2013. http://health.gov.ie/wp-content/uploads/2014/03/key\_trends_2013.pdf

[7] Thompson S, Jowett M, Mladovsky P, Evetovits T, Figueras J, Nolan A, Normand C (2012). **Health system responses to financial pressures in Ireland: policy options in an international context**. Technical report, European Observatory on Health Systems and Policies Brussels.

[8] Briggs AD (2013). **How changes to irish healthcare financing are affecting universal health coverage**. Health Policy.

[9] McDaid D, Wiley M, Maresso A, Mossialos E (2009). **Health care systems in transition: Ireland**. Copenhagen: European Observatory on Health Systems and Policies.

[10] Ryan Pm (2011). **Transforming primary care in Ireland: information, incentives, and provider capabilities**. Centre for Health Policy and Management Working Paper (01),.

[11] Arrow KJ (1963). **Uncertainty and the welfare economics of medical care**. Am Econ Rev.

[12] Van de Ven WPMM, Schut FT (2008). **Universal mandatory health insurance in the Netherlands: a model for the United States?**. Health Aff.

[13] Ryan P, Thomas S, Normand C (2009). **Translating Dutch: challenges and opportunities in reforming health financing in Ireland**. Ir J Med Sci.

[14] Schut F, van de Ven W (2011). **Health care reform in the Netherlands: the fairest of all?**. J Health Serv Res Policy.

[15] Gaynor M, Town R, Mcguire TG, Pauly MV, Baros PP (2011). **Competition in health care markets**. Handbook of Health Economics.

[16] Glied S (2000). **Managed care**. Handbook Health Econ.

[17] Okma KG, Crivelli L (2013). **Swiss and dutch “consumer-driven health care”: Ideal model or reality?**. Health Policy.

[18] NZa (2014). **Monitor Zorginkoop**. NZa;.

[19] **The path to universal healthcare: White paper on universal health insurance** *Department of Health,* 2014. http://www.imo.ie/policy-international-affair/overview/White-paper-on-Universal-Health-Insurance.pdf

[20] Mulholland P (2011). The Private Healthcare Battle.

[21] **Horizontal Merger Guidelines 2010** *US Department of Justice and Federal Trade Commission* 2013. http://www.justice.gov/atr/public/guidelines/hmg-2010.html

[22] **Improving Healthcare: a Dose of Competition***a Report by the Federal Trade Commission and the Department of Justice* 2005. http://www.ftc.gov/reports/improving-health-care-dose-competition-report-federal-trade-commission-department-justice 16613355

[23] Turner B, Shinnick E (2013). **Community rating in the absence of risk equalisation: lessons from the irish private health insurance market**. Health Econ Pol Law.

[24] Kenny MA (2011). it Harney Under Pressure to Publish Vhi Finance Audit.

[25] Mossialos E, Lear J (2012). **Balancing economic freedom against social policy principles: Ec competition law and national health systems**. Health Policy.

[26] NZa (2012). Marktscan Zorgverzekeringsmarkt. Weergave van de markt 2008–2012.

[27] Leu RE, Fund C (2009). The Swiss and Dutch Health Insurance Systems: Universal Coverage and Regulated Competitive Insurance Markets.

[28] NZa (2013). Marktscan Zorgerzekeringsmarkt 2013.

[29] Dafny LS (2010). **Are health insurance markets competitive?**. Am Econ Rev.

[30] Boonen LHHM, Schut FT (2011). **Preferred providers and the credible commitment problem in health insurance: first experiences with the implementation of managed competition in the Dutch health care system**. Health Econ Pol Law.

[31] Halbersma R, Mikkers MC, Motchenkova E, Seinen I (2011). **Market structure and hospital–insurer bargaining in the Netherlands**. Eur J Health Econ.

[32] Gaynor M (2012). **Reform, Competition, and Policy in Hospital Markets**. Submission to the Organisation for Economic Co-operation and Development Roundtable on Competition in Hospital Markets.

[33] Chernew ME, Wodchis WP, Scanlon DP, McLaughlin CG (2004). **Overlap in HMO physician networks**. Health Aff.

[34] Dranove D, Lindrooth R, White WD, Zwanziger J (2008). **Is the impact of managed care on hospital prices decreasing?**. J Health Econ.

[35] Bal R, Zuiderent-Jerak T (2011). **The practice of markets in Dutch health care: are we drinking from the same glass?**. Health Econ Pol Law.

[36] Maarse H, Paulus A (2011). **The politics of health-care reform in the Netherlands since 2006**. Health Econ Pol Law.

[37] Halbersma RS, Mikkers MC, Kerstholt W (2009). **Marktafbakening en marktmacht in de zorgsector**. Markt en Mededinging.

[38] Maarse H (2009). **Health care reform-more evaluation results**. Health Policy Monitor,.

[39] Kalogirou S, Foley R (2006). **Health, Place and Hanly: modelling accessibility to hospitals in Ireland**. Ir Geogr.

[40] Schafer W, Kroneman M, Boerma W, van den Berg M, Westert G, Deville W, van Ginneken E (2010). **The Netherlands: health system review**. Health Syst Transit.

[41] Wren MA (2003). Unhealthy State: Anatomy of A Sick Society.

[42] Thomas S, Normand C, Smith S (2006). Social Health Insurance: Options for Ireland.

[43] **Health in Ireland - Key Trends 2012** *Technical report, Department of Health, Dublin* 2013. http://health.gov.ie/wp-content/uploads/2014/03/KeyTrends_2012.pdf

[44] Kemp RG, Kersten N, Severijnen AM (2012). **Price effects of dutch hospital mergers: an ex-post assessment of hip surgery**. De Economist.

[45] Akosa-Antwi Y, Gaynor M, Vogt W (2007). **Evaluation of Merger Simulation: Evidence from the Hospital Market in California**. Technical report mimeo Carnegie Mellon University.

[46] Canoy MFM, Mikkers MC (2012). **Zorginkoop: de lakmoesproef voor het nieuwe stelsel. Een economisch gezonde gezondheidszorg**. Preadviezen van de Koninklijke Vereniging voor de Staathuishoudkunde.

[47] Douven MRR, Mosca I (2012). **The Effect of Physician Fees and Density Differences on Regional Variation in Hospital Treatments**. CPB discussion Paper.

[48] Wennberg JE (2010). Tracking Medicine: A Researcher’s Quest to Understand Health Care.

[49] Brick A, Nolan A, O’Reilly J, Smith S (2010). Resource Allocation, Financing and Sustainability in Health Care.

[50] Busse R, Geissler A, Quentin W, Wiley M (2011). Diagnosis-Related Groups in Europe: Moving Towards Transparency, Efficiency and Quality in Hospitals.

[51] **Money follows the patient** *Technical report, Department of Health, Dublin* 2013. health.gov.ie

[52] **National Service Plan** *HSE; Health Service Executive; Dublin, Ireland;* 2013. http://www.hse.ie/eng/services/Publications/corporate/NSP2013.pdf

[53] Cutler DM, Zeckhauser RJ (1997). **Adverse selection in health insurance**. Technical report, National Bureau of Economic Research.

[54] **Report of The Health Insurance Authority to the Minister for Health, in accordance with Section 7E(1)(b) of the Health Insurance Acts, 1994–2009, 2011** *Health Insurance Authority, Dublin* 2011. http://health.gov.ie/blog/publications/report-of-the-hia-to-the-minister-for-health-in-accordance-with-section-7e1b-of-the-health-insurance-acts-1994-2009-redacted-version/

[55] Thomas S, Ryan P, Normand C (2010). Effective Foundations for the Financing and Organisation of Social Health Insurance in Ireland.

[56] Van de Ven WPMM (2011). **Risk adjustment and risk equalization: what needs to be done?**. Health Econ Pol Law.

[57] Brabers AE, Reitsma-van Rooijen M, de Jong JD (2012). **The dutch health insurance system: mostly competition on price rather than quality of care**. Eurohealth.

[58] Eireann D (2012). **Health insurance (amendment) bill 2012**.

[59] Houses of the Oireachtas (2013). **Health Identifiers Bill 2013**.

[60] Health Service Executive and Department of Health (2013). **eHealth Strategy for Ireland**.

[61] Smith S (2010). **The irish ‘health basket’: a basket case?**. Eur J Health Econ.

[62] Zegers M, De Bruijne M, Wagner C, Hoonhout L, Waaijman R, Smits M, Hout F, Zwaan L, Christiaans-Dingelhoff I, Timmermans D, Groenewegen PP, Van der Wal G (2009). **Adverse events and potentially preventable deaths in dutch hospitals: results of a retrospective patient record review study**. Qual Health Care.

[63] Merten H, Zegers M, De Bruijne MC, Wagner C (2013). **Scale, nature, preventability and causes of adverse events in hospitalised older patients**. Age Ageing.

[64] Westert GP, Faber M (2011). **Commentary: the dutch approach to unwarranted medical practice variation**. BMJ.

[65] **National standards for safer better healthcare** *Health Information and Quality Authority, Dublin* 2012. http://www.hiqa.ie

[66] **Coding notes - hospital acquired diagnosis hadx** *ESRI, HIPE & NPRS Unit, Dublin* 2013. http://www.esri.ie

[67] Damman OC, van den Hengel YK, van Loon AJM, Rademakers J: **An international comparison of web-based reporting about health care quality: content analysis****.** *J Med Internet Res* 2010.,**12**(2)**:** http://www.ncbi.nlm.nih.gov/pmc/articles/PMC2885782/10.2196/jmir.1191 PMC2885782 20439252

[68] Bijlsma MJ, Koning PW, Shestalova V (2013). **The effect of competition on process and outcome quality of hospital care in the netherlands**. De Economist.

[69] Lako CJ, Rosenau P (2009). **Demand-driven care and hospital choice. Dutch health policy toward demand-driven care: results from a survey into hospital choice**. Health Care Anal.

[70] Bartelink S, Lako C (2012). **How do the dutch choose their hospitals? Results from a survey among 311 patients**. J Health Med Informat S.

[71] Lako CJ, Rosenau P, Daw C (2011). **Switching health insurance plans: results from a health survey**. Health Care Anal.

[72] Davies HT (2003). **The economic evolution of american health care: From marcus welby to managed care**. J R Soc Med.

[73] Mason A, Street A (2006). **Publishing outcome data: is it an effective approach?**. J Eval Clin Pract.

[74] van Oostrom SH, Picavet HSJ, van Gelder BM, Lemmens LC, Hoeymans N, van Dijk CE, Verheij RA, Schellevis FG, Baan CA (2012). **Multimorbidity and comorbidity in the dutch population–data from general practices**. BMC Public Health.

[75] Glazer J, McGuire TG (2002). **Multiple payers, commonality and free-riding in health care: Medicare and private payers**. J Health Econ.

[76] Chen HF, Bazzoli GJ, Harless DW (2010). **Is quality of cardiac hospital care a public or private good?**. Medical Care.

[77] Pronovost P, Vohr E (2010). Safe Patients, Smart Hospitals: How One doctor’s Checklist Can Help Us Change Health Care from the Inside Out.

[78] Enthoven AC (1993). **The history and principles of managed competition**. Health Aff.

[79] Kronick R, Goodman DC, Wennberg J, Wagner E (1993). **The marketplace in health care reform–the demographic limitations of managed competition**. N Engl J Med.

[80] Cebul R, Herschman R, Rebitzer JB, Taylor LJ, Votruba M (2007). Employer-based insurance markets and investments in health.

[81] Herring B (2010). **Suboptimal provision of preventive healthcare due to expected enrollee turnover among private insurers**. Health Econ.

[82] Kanters TA, Brouwer WB, van Vliet RC, van Baal PH, Polder JJ (2013). **A new prevention paradox: The trade-off between reducing incentives for risk selection and increasing the incentives for prevention for health insurers**. Soc Sci Med.

[83] Faber MJ, Burgers JS, Westert GP (2012). **A sustainable primary care system: Lessons from the netherlands**. J Ambul Care Manag.

[84] van den Gronden JW, Szyszczak E, Neergaard U, Krajewski M (2011). Experiences from the Netherlands; the Application of Competition Rules in Health Care.

[CR85] The pre-publication history for this paper can be accessed here:http://www.biomedcentral.com/1472-6963/14/442/prepub

